# Delayed recovery from paralysis associated with plasma cholinesterase deficiency

**DOI:** 10.1186/s40064-016-3561-y

**Published:** 2016-10-28

**Authors:** Wenqin Zhou, Sheng Lv

**Affiliations:** 1 Department of Anesthesiology, West China Second University Hospital, Sichuan University, Chengdu, 610041 China; 2 Key Laboratory of Obstetric Gynecologic and Pediatric Diseases and Birth Defects, Ministry of Education, Chengdu, 610041 Sichuan China

## Abstract

**Introduction:**

This case was to describe a patient presented a 6 h length of apnea associated with low cholinesterase activity.

**Case description:**

A 32 years old female patient (body weight 50 kg, height 160 cm) was admitted to the hospital for laparoscopy combined with hysteroscopy exploration. The preoperative interrogation revealed no significant personal or family history of adverse reaction to anesthetics. The patient was healthy, with no chronic or systemic disease. ASA classification is I. We performed a general anesthesia with intubation to the patient. Succinylcholine 100 mg was administered in anesthesia induction. After intubation, cisatracurium 3 mg and 3% sevoflurane were used for anesthesia maintenance. The patient had been mostly unresponsive to external stimuli for 10 min since the end of the operation. Six hours after operation, the patient had totally recovered from paralysis and tracheal tube was extubated. The plasma cholinesterase test showed 291 U/L, significantly below normal (4650–10,440 U/L). Three days after operation, the patient was discharged from hospital with no special discomfort.

**Discussion and evaluation:**

Reduced plasma cholinesterase activity may occur as a result of inherited, acquired defects or iatrogenic causes. If the acquired defects are excluded, low BChE activity is usually considered to be caused by mutations in butyrylcholinesterase gene (BCHE). 80% of the patients experiencing prolonged neuromuscular blockade following mivacurium have butyrylcholinesteraseen enzyme (BChE) deficiency of genetic origin. The novel mutation of BChE gene is usually associated with the ethnic of the patients. There is no specific treatment for butyrylcholinesterase deficiency and the mainstream is to maintain ventilatory support until succinlcholine is metabolized out of the myoneural junction and neuromuscular function recovers. Transfusion of fresh frozen plasma is also viable.

**Conclusions:**

Plasma cholinesterase deficiency is a genetic or acquired condition. The most obvious feature of this genetic variants is prolonged recovery from paralysis in which administrated with succinylcholine or mivacurium. Once this is suspected, a laboratory test is important. There is no specific treatment for plasma cholinesterase deficiency. The best and safest way is to let the patient recover spontaneously. Mechanical ventilation support is important.

## Case report

Cholinesterase is produced by liver and released into plasma. It is involved in the hydrolysis of ester bonds in muscle relaxants like succinylcholine and several other drugs. It is reported in the case prolonged recovery from paralysis caused by cholinesterase deficiency in a healthy young woman who was not diagnosed so preoperatively.

A 32-year-old female patient (weight 50 kg, height 160 cm), ASA I, was admitted to our hospital for laparoscopy combined with hysteroscopy exploration. The preoperative evaluation revealed no significant personal or family history for adverse reaction to anesthetics. Her preoperative tests were normal, including blood routine examination, liver and kidney function, ECG and chest radiograph. A general anesthesia was scheduled for this patient. Midazolam 2 mg, fentanyl 0.2 mg, propofol 100 mg and succinylcholine 100 mg were administered for anesthesia induction. After intubation, cisatracurium 3 mg and 3% sevoflurane were used for anesthesia maintenance. The operation finished successfully within 45 min. The vital signs had been stable during the operation.

The patient was mostly unresponsive to external stimuli for 10 min since the end of the operation. So we gave her neostigmine (1 mg) and atropine (0.5 mg) to reverse the neuromuscular blockade. As our hospital did not have a peripheral nerve stimulator, it was hard to accurately assess the extent of neuromuscular blockade. We had to keep stimulating the patient and observed the reaction. But the patient was still unconscious 30 min after the operation. The artery blood gas analysis showed slight respiratory acidosis (pH 7.308, PCO_2_: 48.8 mmHg, PO_2_: 480 mmHg, BE −2 mmol/L, ${\text{HCO}}_{3}^{ - }$: 24.6 mmol/L, SPO_2_ 100%, Na^+^: 140 mmol/L, K^+^: 3.8 mmol/L, iCa^2+^: 1.13 mmol/L, Hct: 0.34, Hb: 118 g/L). We suspected the neuromuscular blockade might not be reversed.

One hour and 25 minutes after operation, the patient coughed when endotracheal tube suction. Two hours and fifteen minutes after operation, the tidal volume of the patient still could not reach the standard. Thus, we delivered her into ICU with tracheal tube. Before delivery, the venous blood samples were sent to test plasma cholinesterase level. Six hours after operation, the patient had totally recovered from paralysis and tracheal tube was extubated. The plasma cholinesterase test showed 291 U/L, far below the standard (4650–10,440 U/L). Three days after operation, the patient was discharged home without special discomfort.

## Discussion

When prolonged neuromuscular blockade happens, a systematic review was necessary to perform. First of all, which kind of neuromuscular relaxant was used? Non-depolarizing or depolarizing, the total dosage, the time of use. In this case, we had used succinylcholine as muscle relaxant during induction, followed with cisatracurium 3 mg. Succinylcholine is commonly used in the surgical rapid-sequence intubation because of its quick effect and short duration of action (Leadingbam 2007). The onset of muscle relaxant after intravenous injection occurs in about 1 min. After a single intubating dose, a complete recovery normally occurs in about 10–12 min. It is hydrolyzed by plasma cholinesterase (Dell 1996). Reduced plasma cholinesterase activity may occur as a result of inherited, acquired defects or iatrogenic causes (Soliday 2010). Low BChE activity is associated with hepatic and renal diseases. For example, oral contraceptives may decrease the level of BChE, and metoclopramide may lead to low BChE activity. If the acquired defects are excluded, low BChE activity is usually considered to be caused by mutations in butyrylcholineesterase gene (BCHE) (Wichmann et al. 2016). Eight percentage of the patients experiencing prolonged neuromuscular blockade following mivacurium have butyrylcholineesterase enzyme (BChE) deficiency of genetic origin (Ok et al. 2013). BChE deficiency is an autosomal recessive trait. The most important variants are the atypical (A-variants), the Kalow (K-variants), the fluoride (F-variants) and the silent variants. In the White population, the A- and K-variants are the most common. There was little information on genetic variants of BChE in Chinese population (Lockridge 2015). The novel mutation of BChE gene is usually associated with the ethnic of the patients. In this case, the patient was born in Sichuan, China, Han ethnic group. We also measured the plasma cholinesterase of the patient’s sister. It showed that her plasma cholinesterase was significantly below normal. It suggests that the family carries a genetic variant of BChE.

There is no specific treatment for butyrylcholineesterase deficiency and the mainstream is to maintain ventilatory support until succinylcholine is metabolized out of the myoneural junction and neuromuscular function recovers. Transfusion of fresh frozen plasma is also viable.

Once a patient was suspected with plasma cholinesterase deficiency, the following measures could be very important: first of all, mechanical ventilation with sedation is necessary until succinylcholine is completely metabolized; secondly, succinylcholine must be avoided; thirdly, test of plasma cholinesterase level of the patient and his/her family members is need; fourthly, marking the patient as plasma cholinesterase deficiency patient; In the end, reviewing the family history of plasma cholinesterase is important.

The limitation of this case is we did not use the neuromuscular stimulator to measure the neuromuscular blockade because our hospital did not have it. However, it is very important to use a neuromuscular stimulator when muscle relaxant was used. Lack of neuromuscular monitoring may increase the risk of distressing awareness, which may lead to post traumatic stress disorder (PTSD) (Thomsen et al. 2015). The patient should be sufficiently sedated until recovery, if the hospital did not have a neuromuscular stimulator.

## Summary

Plasma cholinesterase deficiency is a genetic or acquired condition.

The most obvious feature of this disease is prolonged recovery from paralysis in which administrated with succinylcholine or mivacurium. Once this disease is suspected, a laboratory test is important. Furthermore, the patient’s family should be tested for BChE deficiency. There is no specific treatment for plasma cholinesterase deficiency. The best and safest way is to let the patient recover spontaneously. Mechanical ventilation support with sufficiently sedated is important.
